# Erectile dysfunction and the risk of prostate cancer

**DOI:** 10.18632/oncotarget.17082

**Published:** 2017-04-13

**Authors:** Wei-Yu Lin, Ying-Hsu Chang, Cheng-Li Lin, Chia-Hung Kao, Hsi-Chin Wu

**Affiliations:** ^1^ Division of Urology, Department of Surgery, Chang Gung Memorial Hospital, Gia-Yi, Taiwan; ^2^ Chang Gung University of Science and Technology, Chia-Yi, Taiwan; ^3^ Department of Medicine, Chang Gung University, Taoyuan, Taiwan; ^4^ Division of Urology, Department of Surgery, Chang Gung Memorial Hospital, LinKo, Taiwan; ^5^ Management Office for Health Data, China Medical University Hospital, Taichung, Taiwan; ^6^ School of Medicine, China Medical University, Taichung, Taiwan; ^7^ Graduate Institute of Clinical Medical Science, College of Medicine, China Medical University, Taichung, Taiwan; ^8^ Department of Nuclear Medicine and PET Center, China Medical University Hospital, Taichung, Taiwan; ^9^ Department of Bioinformatics and Medical Engineering, Asia University, Taichung, Taiwan; ^10^ Department of Urology, China Medical University Hospital, Taichung, Taiwan; ^11^ Graduate Institute of Clinical Medical Science and School of Medicine, College of Medicine, China Medical University, Taichung, Taiwan

**Keywords:** Prostate cancer (PCa), erectile dysfunction (ED), Cohort study, National Health Insurance Research Database, malignancy

## Abstract

**Background:**

Prostate cancer (PCa) is the most commonly diagnosed malignancy and the third leading cause of cancer death among men in developed countries. Because some risk factors are common between erectile dysfunction (ED) and PCa, we investigated the association between ED and subsequent PCa.

**Methods:**

This nationwide population-based cohort study used data from the Taiwan National Health Insurance Research Database for the period 2000–2010. We identified patients newly diagnosed with ED by using codes from the International Classification of Diseases, Ninth Revision, Clinical Modification.

**Results:**

In total, 5858 and 23432 patients were enrolled in the ED and non-ED cohorts, respectively. After adjustment for age, sex, and comorbidities, the overall incidence densities of PCa were significantly higher in the ED cohort than in the non-ED cohort, with an adjusted hazard ratio (aHR) of 1.19. The age-specific relative risk of PCa was significantly higher for all age groups in the ED cohort than in the non-ED cohort. Compared with patients without ED, those with organic ED had a 1.27-fold higher risk of PCa.

**Conclusion:**

ED is a harbinger of PCa in some men. Physicians should consider the possibility of occult PCa in patients with ED regardless of age and comorbidities.

## INTRODUCTION

Prostate cancer (PCa) is the most commonly diagnosed malignancy and the third leading cause of cancer death among men in developed countries [1]. In the United States alone, an estimated 242 000 men were diagnosed with PCa in 2012, and 28,000 of them will die from the disease [1, 2]. In Asia, although a trend of stage migration to the earlier stage has been observed in some countries, a relatively high rate of advanced PCa exists compared with the rates in the United States and Europe. This difference is mainly attributed to the lack of mass screenings [3–6].

Despite the high incidence and mortality of PCa, few risk factors have been identified for the disease. Among those that have are older age, smoking, and obesity [7]. Further investigation of the risk factors and their associated mechanisms of carcinogenesis is crucial to develop strategies for preventing and treating PCa [8].

Sexual activity is hypothesized as affecting prostate carcinogenesis, though the association between ejaculatory frequency and subsequent risk of PCa is controversial [9, 10]. A previous study revealed that compared with men with erectile dysfunction (ED) who were not treated with phosphodiesterase type 5 inhibitors (PDE5i), men with ED who were treated with PDE-5i tended to exhibit a lower likelihood of being diagnosed with PCa because they were expected to exhibit higher ejaculation frequency than those not treated with PDE5i [11].

The risk of ED is related to many factors including age, smoking, diabetes, heart disease, depression, and hypertension [12–14].

PCa and ED share some common risk factors, including age, smoking, and obesity. In addition, ED is correlated with advanced PCa [15]. Furthermore, a previous study suggested that preoperative ED is a surrogate for adverse PCa outcomes following radical prostatectomy [16]. We postulate that ED is a sentinel symptom in patients with occult PCa. Thus, to examine this hypothesis, we performed a large-scale nationwide controlled cohort study in Taiwan to investigate whether ED increases the risk of PCa [17].

## RESULTS

**Figure 1:**
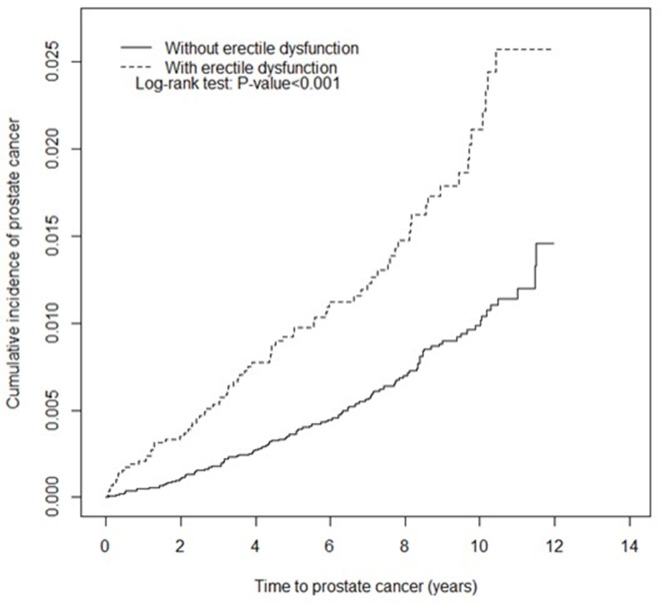
Kaplan–Meier curve of the cumulative incidence of prostate cancer in the cohorts of patients with and without erectile dysfunction

We included 5857 patients in the ED cohort and 5857 patients in the non-ED cohort. The age, occupation, urbanization level, and comorbidities distributions of the cohorts were similar (Table 1). The largest age subgroup of the men in both cohorts was ≤ 49 years old (39.3% vs. 39.9%), and most of the men were white-collar workers (55.0% vs 56.3%) and lived in an urbanized area (66.5% vs 67.6%). The mean frequency of medical visits/per year was 22.1 (SD=18.1) and 21.5 (SD=20.6) years for the ED and non- ED cohorts, respectively. The mean age of the ED and non-ED cohorts was 53.6 and 53.1 years, respectively. The cumulative incidence curves of PCa revealed that the ED cohort had a significantly higher risk of PCa than did the non-ED cohort (*P* < .001, log-rank test) (Figure 1).

**Table 1:** Comparison of demographic characteristics and comorbidity of patients with ED and controls

	Erectile dysfunction (N =5857)		Control (N =5857)		p-value
	n	%	n	%	
Age, year					0.04
≤ 49	2300	39.3	2337	39.9	
50-64	2273	38.8	2149	36.7	
≥65	1284	21.9	1371	23.4	
Mean (SD)^#^	53.6	13.6	53.1	14.4	0.06
Frequency of medical visits/per year	22.1	18.1	21.5	20.6	0.001
Occupation					0.23
White collar	3219	55.0	3289	56.3	
Blue collar	1617	27.6	1539	26.3	
Others^‡^	1021	17.4	1019	17.4	
Urbanization level^†^					0.05
1 (highest)	2038	34.8	2098	35.8	
2	1854	31.7	1871	31.9	
3	1014	17.3	1043	17.8	
4(lowest)	951	16.2	845	14.4	
Comorbidity					
Hyperlipidemia	1814	31.0	1837	31.4	0.65
Diabetes	923	15.8	968	16.5	0.26
Hypertension	2375	40.6	2386	40.7	0.84
Urinary stones	508	8.67	533	9.10	0.42
Urinary tract infection	303	5.17	274	4.68	0.22
Coronary artery disease	1341	22.9	1310	22.4	0.49
Depression	470	8.02	396	6.76	0.01
Prostate cancer screen					
Prostate biopsy	107	1.83	97	1.66	0.48
TURP	155	2.65	146	2.49	0.60

Chi-square test compared to total SD; ^#^*t* test

NTD, New Taiwan dollar.

^†^: The urbanization level was categorized into 4 levels according to the population density of the residential area, with level 1 indicating the highest urbanization and level 4 indicating the least urbanization.

^‡^: Other occupation categories included primarily retired, unemployed, and low-income populations.

The overall incidence densities of PCa were significantly higher in the ED cohort than in the non-ED cohort (2.02 vs 1.63 per 1000 person-y), with an adjusted HR (aHR) of 1.19 (95% CI = 1.08-1.31) after controlling for age; frequency of medical visits/per year; occupation category; urbanization level; comorbidities of hyperlipidemia, diabetes, hypertension, urinary stones, urinary tract infection, obesity, anxiety, and depression; and PCa screening strategies of prostate biopsy and TURP (Table 2). The age-specific relative risk of PCa was higher for age <64 y groups (aHR = 1.49, 95% CI = 1.32-1.69) in the ED cohort than in the non-ED cohort. The occupation category-specific analyses showed that the patients in the ED cohort had a significantly higher risk of PCa than did those in the non-ED cohort for white-collar workers. Patients in the ED cohort had a significantly higher risk of PCa than did those in the non-ED cohort for 2^th^ or 3^th^ urbanization level. The relative risk of PCa for the ED cohort compared with the non-ED cohort was significantly higher for both men without comorbidity (aHR = 1.95, 95% CI = 1.65-2.30). In both cohorts, undergoing prostate biopsy or TURP was associated with an increased incidence of PCa. Among the patients without prostate biopsy, men with ED had a higher risk of PCa than did those without ED (aHR = 1.43, 95% CI = 1.28-1.60). Similar results were observed for the patients without TURP; the ED cohort had a 1.53-fold higher risk of PCa than did the non-ED cohort (95% CI = 1.38-1.70). Compared with the patients without ED, those with organic ED had a 1.27-fold higher risk of PCa (95% CI = 1.15-1.40) (Table 3).

**Table 2:** Comparison of the incidence densities and hazard ratios of prostate cancer of men with and without erectile dysfunction stratified by demographic characteristics, comorbidity, prostate cancer screening, and medication

	Erectile dysfunction							
	Yes			No				
	Event	PY	Rate^#^	Event	PY	Rate^#^	Crude HR (95% CI)	Adjusted HR^†^ (95% CI)
All	76	37603	2.02	59	36221	1.63	1.24(1.10,1.39)***	1.19(1.08, 1.31)***
Age								
≤ 64	28	29092	0.96	17	28218	0.60	1.60(1.38, 1.84)***	1.49(1.32, 1.69)***
≧65	48	8511	5.64	42	8004	5.25	1.08(0.86, 1.34)	0.99(0.81, 1.20)
Occupation								
White collar	36	19998	1.80	25	20534	1.22	1.48(1.26, 1.74)***	1.41(1.23, 1.61)***
Blue collar	15	10428	1.44	19	9508	2.00	0.71(0.36, 1.39)	0.97(0.49, 1.94)
Others^‡^	25	7178	3.48	15	6179	2.43	1.44(1.10, 1.88)**	1.05(0.83, 1.33)
Urbanization level								
1 (highest)	27	13139	2.06	24	12926	1.86	1.11(0.91, 1.35)	0.92(0.79, 1.08)
2	26	11805	2.20	14	11796	1.19	1.86(1.49, 2.31)***	1.35(1.12, 1.63)**
3	12	6546	1.83	9	6325	1.42	1.29(0.98, 1.69)	1.68(1.33, 2.12)***
4(lowest)	11	6113	1.80	12	5175	2.32	0.78(0.58, 1.04)	1.00(0.78, 1.27)
Comorbidity^‡^								
No	12	13641	0.88	7	16057	0.44	2.02(1.64, 2.49)***	1.95(1.65, 2.30)***
Yes	64	23962	2.67	52	20165	2.58	1.04(0.90, 1.20)	1.10(0.98, 1.25)
Prostate cancer screen								
Prostate biopsy								
No	47	36843	1.28	31	35516	0.87	1.46(1.29, 1.66)***	1.43(1.28, 1.60)***
Yes	29	760	38.1	28	706	39.7	0.96(0.57, 1.63)	0.88(0.52, 1.49)
TURP								
No	61	36388	1.68	36	35109	1.03	1.64(1.44, 1.85)***	1.53(1.38, 1.70)***
Yes	15	1216	12.3	23	1112	20.7	0.60(0.35, 1.02)	0.59(0.34, 1.01)

Rate^#^, incidence rate per 1000 person-years; Crude HR, relative hazard ratio

Adjusted HR^†^: Multivariate analysis with adjustment for age; frequency of medical visits/per year; occupation; urbanization level; comorbidities of hyperlipidemia, diabetes, hypertension, urinary stones, urinary tract infection, obesity, anxiety, and depression; and prostate cancer screening strategies of prostate biopsy and TURP

Comorbidity^‡^: Only one comorbidity (namely hyperlipidemia, diabetes, hypertension, urinary stones, urinary tract infection, coronary artery disease, and depression) classified as the comorbidity group. *P* < .05, **P < .01, ***P < .001

**Table 3:** Comparison of the incidence and hazard ratios of prostate cancer stratified by subtypes of erectile dysfunction

Variables	N	Event	Rate^#^	Crude HR (95% CI)	Adjusted HR^†^ (95% CI)
Without erectile dysfunction	5857	59	1.63	1(Reference)	1(Reference)
Erectile dysfunction					
Psychogenic ED	579	3	0.76	0.44(0.14, 1.43)	0.43(0.13, 1.37)
Organic ED	5278	73	2.17	1.33(1.18, 1.50)***	1.27(1.15, 1.40)***

Rate^#^, incidence rate per 1000 person-years; Crude HR, relative hazard ratio

Adjusted HR^†^: Multivariate analysis adjusted for age; frequency of medical visits/per year; occupation; urbanization level; comorbidities of hyperlipidemia, diabetes, hypertension, urinary stones, urinary tract infection, obesity, anxiety, and depression; and prostate cancer screening strategies of prostate biopsy and TURP ***P < .001

The aHR of PCa slightly decreased as the follow-up duration increased (Table 4). Throughout the follow-up period, the ED cohort exhibited a higher risk of PCa than did the non-ED cohort, except for follow-up period>5.

**Table 4:** Trends of prostate cancer risks stratified by follow-up duration

	Erectile dysfunction							
	Yes			No				
Follow-up time, years	Event	PY	Rate^#^	Event	PY	Rate^#^	Crude HR (95% CI)	Adjusted HR^†^ (95% CI)
≤1	12	5824	2.06	5	5800	0.86	2.39(2.04, 2.80)***	2.33(2.03, 2.67)***
2-3	17	10383	1.64	11	10227	1.08	1.52(1.32, 1.75)***	1.48(.30, 1.67)***
4-5	17	8434	2.02	13	8159	1.59	1.27(1.09, 1.47)**	1.18(1.04, 1.34)**
>5	30	12963	2.31	30	12035	2.49	0.93(0.80, 1.08)	0.90(0.79, 1.02)

Rate^#^, incidence rate per 10,000 person-years; Crude HR, relative hazard ratio

Adjusted HR^†^: Multivariate analysis including age; frequency of medical visits/per year; occupation; urbanization level; comorbidities of hyperlipidemia, diabetes, hypertension, urinary stones, urinary tract infection, obesity, anxiety, and depression; and prostate cancer screening strategies of prostate biopsy and TURP **P < .01, ***P < .001

## DISCUSSION

Our data suggest that after adjustment for potential confounding factors, the risk of PCa was significantly higher in men with ED than in those without ED. PCa is the third leading cause of cancer death among men in developed countries [2, 18]. In Asia, because of the lack of mass screenings, a high rate of advanced PCa is detected compared with the rate in the United States and Europe [4–6]. Hence, the identification of a predictive symptom or finding would enable even earlier intervention, possibly further reducing morbidity and mortality caused by the disease [14, 19]. Such a predictor would be particularly useful for men who do not receive regular PCa assessments.

This study revealed that compared with men without ED, those with ED exhibited a higher risk of subsequent PCa, regardless of age, comorbidities, and urbanization level. The risk of PCa was also higher in white-collar workers. The risk of PCa in our study population is significantly higher than that in the general population, even for a follow-up duration of more than 5 years. This finding may be partially attributable to the higher rate of prostate-related surgery (prostate biopsy plus TURP) in patients with ED (Table 1), which may result from more frequent contact with health care providers, leading to increased PCa screening and detection.

In order to decrease the surveillance bias, which resulted from more frequent contact with health providers because of other co-morbidities, We re-created a non-ED cohort well matched for age, frequency of medical visits/per year, occupation, urbanization level, index year and comorbidities including hyperlipidemia, diabetes, hypertension, urinary stones, urinary tract infection, coronary artery disease, depression, prostate cancer screen, prostate biopsy, and TURP (Table 1). With this newly selected non-ED cohort, the associations between ED and PCa remain significant, although the HR decreases from 1.41 to 1.19 (Table 2).

The relationship between ED and subsequent diagnosis of PCa has not been investigated. Most studies have investigated the prevalence of ED before curative treatments for PCa through retrospective analysis [15, 16, 20]. To the best of our knowledge, this is the first large-scale nationwide cohort study to assess the relationship between ED and the risk of subsequent PCa.

In the present study, 5279 of the 5858 patients with ED (90.1%) had organic ED (Table 3), which includes neurologic, hormonal, arterial, and cavernosal impairment etiologies. In addition, the cultural taboo in Asia against discussing sexuality should be considered [17]. A previous study suggested that ED is correlated with advanced PCa [15]. In the current study, the aHR for PCa in men with organic ED was 1.27 (95% CI = 1.15–1.40) compared with that of men with psychogenic ED (0.43, 95% CI = 0.13-1.37). Notably, among the men who had ED before PCa diagnosis, organic ED was the most common type. This result is different to the finding that a mix of organic and psychogenic ED is a common complication following prostate cancer treatment among men with PCa [15].

Our finding of a positive association between ED and subsequent diagnosis of PCa in follow-up duration of less than 1 year does not support an etiological role for ED in initiating prostate carcinogenesis because PCa tends to be slow growing with a protracted latency period; up to 20–30 years are required before the disease appears clinically [21, 22]. Thus, it is plausible to assume the coexistence of ED and occult PCa rather than initiation of new-onset PCa. Therefore, our results may provide indications to aid clinical physicians in deciding whether to assess the risk of PCa in patients with ED.

On the other side, a high prevalence of ED has been observed in patients with chronic prostatitis/chronic pelvic pain syndrome (CP/CPPS), and a systemic review and meta-analysis confirmed a close association between ED and CP/CPPS [17, 23]. In addition, meta-analyses of case–control studies have reported statistically significant associations between PCa and prostatitis (odds ratio = 1.6) [8, 24, 25]. Therefore, CP/CPPS may play a critical role in contributing to ED and subsequent PCa because pathological studies have shown that inflammation may be involved in the development of PCa [7, 26].

Moreover, compared with men without ED, the aHR for PCa (1.19, 95% CI = 1.08-1.31) was still significantly higher in men with ED, even for a follow-up duration of more than 5 years. We assume that patients with ED might lose the protective effects of frequent ejaculation on the risk of PCa. Such effects include modulated prostate carcinogenesis through the altering of the composition of prostatic fluid; decreased intraprostatic concentration of xenobiotic compounds and chemical carcinogens, which readily accumulate in prostatic fluid [27, 28]; and the reduced development of intraluminal prostatic crystalloids [8, 29].

This study has some limitations. First, no data on the International Index of Erectile Function Questionnaire or penile blood vessel examinations were available regarding the diagnosis of ED in this administrative database. Therefore, we could not analyze the association between the severity of ED and PCa, in contrast to previous studies that used questionnaires to evaluate ED severity [23, 30, 31].

Second, the diagnoses of ED, PCa, and other comorbidities were based on ICD-9-CM codes; thus, misclassification is possible. However, the use of ICD-CM-9 codes for diagnosing chronic diseases has been validated in previous nationwide cohort studies [32–36]. Moreover, the National Health Insurance Administration reviews charts, verifies medical charges, and imposes heavy penalties for inappropriate charges and malpractice. These checks and balances are assumed to ensure accurate coding [34, 37].

Moreover, to further reduce misclassification, the diagnosis of PCa was obtained from the Catastrophic Illness Patient Database, which includes proof of diagnosis by biopsy or tissue pathology. Because of the lack of crucial tumor characteristics in the database, such as stage and grade, we could not analyze the association between ED and PCa stage.

Third, the NHIRD lacks information on some critical ED risk factors such as smoking, obesity, BMI, alcoholism, exercise, and dietary habits. However, we have included hypertension, diabetes, and hyperlipidemia to adjust for the effect of BMI and obesity.

Our data suggest that patients with ED have an approximately 1.24-fold higher risk of subsequent PCa than do patients without ED, even for a follow-up duration of more than 5 years. For patients who initially present with ED, the evaluating physician should screen for PCa, regardless of whether the patient is aged younger or older than 65 years and regardless of the presence or absence of any comorbidity. More research is needed - both regarding the possible biological link and to confirm the findings. In addition future studies should stratify the PCa according to risk class to assess any clinical significance.

## MATERIALS AND METHODS

### Data source

This retrospective cohort study used data from the Longitudinal Health Insurance Database 2000 (LHID2000). The LHID2000 is a subset of the National Health Insurance Research Database (NHIRD) and includes the health claims data of 1 million randomly selected beneficiaries of the Taiwan National Health Insurance (NHI) program for the period 1996–2011. To build the LHID2000, the National Health Research Institutes randomly selected 1 million beneficiaries from the NHIRD between 1996 and 2011. The details of the NHI program and LHID2000 have been described previously [38, 39]. Disease classification in the LHID2000 is based on the International Classification of Diseases, Ninth Revision, Clinical Modification (ICD-9-CM). This study was approved by the Institutional Review Board of China Medical University (CMUH104-REC2-115-CR1).

### Sampled population

To determine the relationship between ED and PCa, we established an ED cohort and a non-ED cohort and observed the incidence of PCa. The ED cohort included men aged ≧20 years with new-onset ED including psychogenic ED (ICD-9-CM code 302.72) and organic ED (ICD-9-CM code 607.84) between January 1, 2000 and December 31, 2010. The index date was the date of first ED diagnosis. To increase the diagnostic validity of ED, only those patients who had been diagnosed with ED at least twice in an outpatient service by urologists were included in the analysis.

The non-ED cohort included men without a history of ED who were randomly selected from the LHID2000. The non-ED cohort was frequency-matched to the ED cohort at a ratio of 1:1 by age (5-y intervals), frequency of medical visits/per year, occupation, urbanization level, index year, and comorbidities including hyperlipidemia, diabetes, hypertension, urinary stones, urinary tract infection, coronary artery disease, depression, prostate cancer screen, prostate biopsy, and TURP. The index date for the patients without ED was the same date as that for matched patients. This study excluded patients with a PCa diagnosis (ICD-9-CM code 185) before the index date. Both cohorts were followed until withdrawal from the NHI program, PCa event occurrence, or December 31, 2011.

### Variables of interest

The sociodemographic variables used in this study were age, occupation category, and urbanization level. The details of occupation category and urbanization level are fully described in a previous study [40, 41]. Pre-existing comorbidities included hyperlipidemia (ICD-9-CM code 272), diabetes (ICD-9-CM code 250), hypertension (ICD-9-CM codes 401-405), urinary stones (ICD-9-CM codes 592.0, 592.1, 594.0, and 594.1), urinary tract infection (ICD-9-CM codes 590 and 595), coronary artery disease (ICD-9-CM codes 410-414), and depression (ICD-9-CM codes 296.2-296.3, 300.4, and 311). Diagnosis of PCa according to tissues obtained from transurethral resection of the prostate (TURP) (ICD-9-procedure code 60.29) and prostate biopsy (ICD-9-procedure codes 60.11 and 60.12).

### Statistical analysis

The demographic and clinical characteristics of the ED and non-ED cohorts, including age, occupation category, urbanization level, comorbidities, and PCa screening strategies, were compared using the Chi-square test. Continuous variables were compared between the ED and non-ED cohorts by using the Student *t* test. Cumulative incidence curves for PCa were plotted using the Kaplan–Meier method, and the curve differences between the cohorts were determined using the log-rank test. We calculated the incidence density rate of PCa (per 1000 person-y) for each cohort. The risk of PCa in the ED cohort compared with that in the non-ED cohort was presented as hazard ratios (HRs) and 95% confidence intervals (CIs) by using univariate and multivariate Cox proportional hazards models. The multivariate Cox models were simultaneously adjusted for age; occupation category; urbanization level; comorbidities of hyperlipidemia, diabetes, hypertension, urinary stones, urinary tract infection, obesity, anxiety, and depression; and PCa screening strategies of prostate biopsy and TURP. All analyses were conducted using SAS statistical software (Version 9.4 for Windows; SAS Institute, Inc., Cary, NC, USA), with statistical significance set at *P* < .05 for a 2-tailed test.
